# Elevated N-Terminal Pro-Brain Natriuretic Peptide Is Associated with Mortality in Tobacco Smokers Independent of Airflow Obstruction

**DOI:** 10.1371/journal.pone.0027416

**Published:** 2011-11-07

**Authors:** Jason A. Stamm, Elizabeth A. Belloli, Yingze Zhang, Jessica Bon, Frank C. Sciurba, Mark T. Gladwin

**Affiliations:** 1 Division of Pulmonary and Critical Care Medicine, Geisinger Medical Center, Danville, Pennsylvania, United States of America; 2 Division of General Internal Medicine, University of Pittsburgh Medical Center, Pittsburgh, Pennsylvania, United States of America; 3 Division of Pulmonary, Allergy, and Critical Care Medicine, University of Pittsburgh Medical Center, Pittsburgh, Pennsylvania, United States of America; 4 Vascular Medicine Institute, University of Pittsburgh Medical Center, Pittsburgh, Pennsylvania, United States of America; Innsbruck Medical University, Austria

## Abstract

**Background:**

Tobacco use is associated with an increased prevalence of cardiovascular disease. N-terminal pro-brain natiuretic peptide (NT-proBNP), a widely available biomarker that is associated with cardiovascular outcomes in other conditions, has not been investigated as a predictor of mortality in tobacco smokers. We hypothesized that NT-proBNP would be an independent prognostic marker in a cohort of well-characterized tobacco smokers without known cardiovascular disease.

**Methods:**

Clinical data from 796 subjects enrolled in two prospective tobacco exposed cohorts was assessed to determine factors associated with elevated NT-proBNP and the relationship of these factors and NT-proBNP with mortality.

**Results:**

Subjects were followed for a median of 562 (IQR 252 – 826) days. Characteristics associated with a NT-proBNP above the median (≥49 pg/mL) were increased age, female gender, and decreased body mass index. By time-to-event analysis, an NT-proBNP above the median (≥49 pg/mL) was a significant predictor of mortality (log rank p = 0.02). By proportional hazard analysis controlling for age, gender, cohort, and severity of airflow obstruction, an elevated NT-proBNP level (≥49 pg/mL) remained an independent predictor of mortality (HR = 2.19, 95% CI 1.07–4.46, p = 0.031).

**Conclusions:**

Elevated NT-proBNP is an independent predictor of mortality in tobacco smokers without known cardiovascular disease, conferring a 2.2 fold increased risk of death. Future studies should assess the ability of this biomarker to guide further diagnostic testing and to direct specific cardiovascular risk reduction inventions that may positively impact quality of life and survival.

## Introduction

Tobacco use is associated with an increased risk of cardiovascular disease, including both coronary artery disease (CAD) and congestive heart failure (CHF) [1], [2], [3], [4]. Moreover, it has been demonstrated that independent of the presence of cardiovascular risk factors, patients with chronic obstructive lung disease (COPD) have double the risk of acute myocardial infarction and over four and one half times the risk of CHF compared to matched controls [5]. Cardiovascular disease is the most common reason for hospital admission in patients with COPD and is a leading cause of death [6], [7], [8], [9].

Brain natriuretic peptide and its precursor, amino terminal pro-brain natiuretic peptide (NT-proBNP), are peptides secreted in response to cardiomyocyte stretch; both have well-characterized diagnostic and prognostic indicators in several cardiovascular disorders [10], [11], [12], [13]. Tobacco smokers have increased NT-proBNP levels compared to non-smokers[14]; in addition, there is evidence that in smokers with COPD elevated NT-proBNP levels are associated with decreased physical activity, exercise tolerance, and latent heart failure [15], [16]. However, NT-proBNP has not been investigated as a predictor of mortality in tobacco smokers without known cardiovascular disease.

We therefore hypothesized and found that elevated NT-proBNP levels independently predict increased mortality in a large cohort of well-characterized tobacco smokers free of prevalent cardiovascular disease. Our results suggest that NT-proBNP can serve as a readily available diagnostic and prognostic screening tool in this at-risk patient population.

## Methods

### Ethics Statement

All subjects provided written informed consent and the study was approved by the University of Pittsburgh Medical Center Institutional Review Board.

### Study Design and Study Population

Subjects were retrospectively analyzed from two large prospective cohorts, the University of Pittsburgh Medical Center COPD Patient Registry and the University of Pittsburgh Specialized Centers in Clinically Oriented Research in COPD cohort and enrolled between the years of 2003 through 2010. Subjects were recruited from the university-based outpatient pulmonary clinic and included a spectrum of obstructive lung disease severity. Inclusion criteria for enrollment in both cohorts were similar and required an age ≥40 years and at least a 10 pack year history of tobacco use; those with any active pulmonary or systemic condition, not related to obstructive lung disease, with significant clinical impact, or significant obesity (body mass index (BMI) ≥36 kg/m^2^), were not enrolled into these cohorts. Exclusion criteria for this analysis included any history of CAD or CHF. Due to the renal clearance of NT-proBNP, renal insufficiency was an additional exclusion criteria (serum creatinine of ≥2.0 mg/dL) [17], [18].

### Clinical Data Collection

Pulmonary function tests were performed at registry enrollment in accordance with published recommendations [19], [20], [21]; post-bronchodilator values were used for all analyses and compared to standard population-derived equations [22], [23]. Dyspnea was scored using the modified Medical Research Council (mMRC) scale [24].

### Plasma Level of NT-proBNP

Venous blood sample was obtained using Vacutainer tube (sodium citrate as anti-coagulator, BD, Franklin Lakes NJ, USA) at registry enrollment. Plasma samples were isolated within 2 hours of collection from the patient and immediately stored at −80°C. NT-proBNP level in plasma was analyzed using a commercially available immunoassay (Roche Elecsys 2010 analyzer, Roche Diagnostics, Manheim, Germany) according to the manufacturer's instruction at the end of the clinical follow up period and after all mortality data were collected [25], [26], [27]. Subjects with NT-proBNP values below the lower detection limit (5 pg/mL) were assigned a value of 5 pg/mL. Laboratory personnel were blinded to survival status of the study participants.

### Definitions

The exclusion criteria of CAD included any subject reported medical diagnosis of angina, myocardial infarction, or coronary revascularization procedure while that of CHF included any subject reported medical diagnosis of heart failure, irrespective of whether reported to be left or right-sided heart failure. Death was ascertained via review of the national Social Security index.

### Statistical Analysis

Baseline characteristics and clinical outcomes were examined via mean ± standard deviation (continuous variables) or as percent (categorical variables). The cohort distribution of NT-proBNP was markedly skewed; there were a large number of patients with low NT-proBNP values and few patients with significantly elevated NT-proBNP levels. Despite transformation the distribution was not normal and therefore the raw values of NT-proBNP were analyzed as a categorical variable dichotomized around the median. Univariate analysis of variables between groups was performed via Student's t tests, Wilcoxon rank-sum tests, or via chi-square tests, as appropriate. Time of survival was calculated from date of registry enrollment until death, with censoring at the date of last query of the Social Security index (March 2010). Multivariable logistic regression analysis using elevated NT-proBNP (above the median) as the dependent outcome was performed to identify relevant predictor variables; variables found to have a p value ≤0.20 in univariate analysis were included in a backward elimination process to arrive at the final model. Survival was assessed via the Kaplan-Meier method and the log-rank test. The strength of the association between mortality and elevated NT-proBNP was assessed via a proportional hazard regression, adjusting for cohort group, age, gender, and severity of obstructive lung disease. Logistic regression model discrimination was assessed via the Hosmer and Lemeshow test. The proportional hazard assumption was assessed via Schoenfeld residuals. A two-sided p-value of ≤0.05 was considered statistically significant. All statistical analyses were performed with Stata, version 10 (StataCorp LP, College Station TX).

## Results

A total of 796 subjects from the two tobacco exposed cohorts were included in this analysis. Baseline clinical characteristics are shown in **Table 1**. The median length of follow-up was 562 days (IQR 252–826 days). 7.4% of subjects had a NT-proBNP value below the lower limit of detection.

**Table 1:** Baseline Characteristics of the Total Study Population.

Characteristic	Total Population
**Demographics**	
Age*(years)	64±8
Gender (% female)	46
BMI (kg/m^2^)	27.6±4.9
Pack Years Tobacco*	51±28
**Disease Characteristics**	
FEV1* (% predicted)	69±28
FVC* (% predicted)	87±19
FEV1/FVC ratio* (percent)	58±18
FRC* (% predicted)	107±31
DLCO* (% predicted)	62±24
GOLD Stage (%)	
0 (At-risk)	35
I	12
II	26
III–IV	27
Oxygen requirement (%)	21
mMRC Dyspnea Score*	2.3±1.5
**NT-proBNP Distribution(pg/mL)**	
Mean ± SD	99±262
Median	49
25^th^ percentile	22
75^th^ percentile	94

*mean ± SD.

*Definitions of abbreviations*: BMI = body mass index; DLCO = diffusion capacity for carbon monoxide; FEV1 = forced expiratory volume in one second; FVC = forced vital capacity; FRC = functional residual capacity; GOLD = Global Initiative for Chronic Obstructive Lung Disease; mMRC = modified Medical Research Council; NT-proBNP = N-terminal pro brain natriuretic peptide.

### Predictors of elevated N-terminal pro-BNP

Correlation analysis between raw NT-proBNP levels and subject characteristics is shown in **Table S1**. Clinical characteristics found to be associated with elevated NT-proBNP (above the median) in univariate analysis are shown in **Table 2**. In multivariable regression modeling incorporating all of those variables with a p value≤0.20, the following variables were found to independently predict an elevated NT-proBNP: increasing age (per decade) (OR 2.1, 95% CI 1.7–2.7, p<0.001), female gender (OR 2.4, 95% CI 1.7–3.4, p<0.001) and decreasing BMI (per kg/m^2^) (OR 0.96, 95% CI 0.93–1.0, p = 0.04).

**Table 2:** Predictors of Elevated N-terminal pro-BNP (≥49 pg/mL).

Variable	Low NT-proBNP	High NT-proBNP	P Value
**N = 796 total** *			
**Demographics**			
Age† (years)	62±7	66±8	<0.001
Gender (% female)	38	54	<0.001
BMI (kg/m^2^) † (n = 676 total)	28.0±4.7	27.2±5.1	0.05
Pack years tobacco†	51±31	50±26	0.57
**Disease Characteristics**			
FEV1† (% predicted)	69±29	69±28	0.84
FVC† (% predicted)	8±18	86±19	0.39
FRC† (% predicted) (n = 575 total)	106±34	108±28	0.34
DLCO† (% predicted) (n = 680 total)	64±24	61±24	0.17
mMRC Dyspnea Score†	2.2±1.5	2.3±1.4	0.21

*for each variable unless otherwise indicated.

† mean ± SD.

*Definitions of abbreviations*: BMI = body mass index; FEV1 = forced expiratory volume in 1 second; FVC = forced vital capacity; FRC = functional residual capacity; DLCO = diffusion capacity for carbon monoxide; mMRC = modifed Medical Research Council.

### Predictors of Mortality

**Figure 1:**
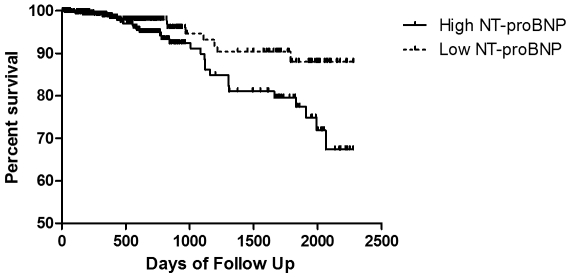
Univariate Survival Analysis. Kaplan-Meier survival curve of those with High (≥49 pg/mL) (solid line) versus Low (<49 pg/mL) (dashed line) NT-proBNP levels. Log-rank test = 0.02. NT-proBNP = N-terminal pro-brain natriuretic peptide.

Overall mortality in the study cohort was 5.3% (n = 42). Survival at specific time points of follow up is shown in **Table S2**. In those patients who died during the study period the median NT-proBNP level was 71 pg/mL (IQR 36–107 pg/mL), compared to a median value of 47 pg/mL (IQR 21–93 pg/mL) in those who remained alive at the end of the study period (p = 0.02). Percent mortality was 7.3% in those with NT-proBNP values above the median (≥49 pg/mL), compared to 3.3% in those with NT-proBNP levels below the median (p = 0.01). Similar results were found when NT-proBNP was analyzed by tertiles (**Table S3**).By time-to-event (Kaplan-Meier) analysis, an NT-proBNP above the median (≥49 pg/mL) was a significant predictor of mortality in the entire study cohort (log rank p = 0.02) (**Figure 1**). Other predictors of mortality in univariate proportional hazard analyses are shown in **Table 3**.

**Table 3:** Clinical Variables and Mortality in Univariate Proportional Hazard Survival Analysis.

	Hazard Ratio	95% Confidence Interval	P Value
**Demographics**			
Age (per decade)	1.46	1.01 – 2.19	0.046
Gender (male)	1.51	0.77 – 2.97	0.23
BMI (kg/m^2^)	0.96	0.90 – 1.02	0.18
Low BMI (≤21 kg/m^2^)	3.56	1.55–8.17	0.003
Pack Years tobacco (per year)	1.00	0.99 – 1.01	0.53
**Laboratory**			
Dichotomized NT-proBNP (≥49 pg/mL)	2.17	1.13 – 4.18	0.02
**Disease Characteristics**			
FEV1 (per decile, % predicted)	0.75	0.65 – 0.85	<0.001
FRC (per decile, % predicted)	1.19	1.10 – 1.29	<0.001
RV/TLC ratio (per decile, %)	1.95	1.38 – 2.76	<0.001
DLCO (per decile, % predicted)	0.57	0.45 – 0.73	<0.001
mMRC Score (per point)	1.71	1.28 – 2.27	<0.001

*Definitions of abbreviations:*BMI = body mass index; DLCO = diffusion capacity for carbon monoxide; FEV1 = forced expiratory volume in 1 second; FRC = functional residual capacity; mMRC = modified Medical Research Council; NT-proBNP = N-terminal pro brain natriuretic peptide; RV/TLC ratio = residual volume to total lung capacity ratio.

In a multivariable proportional hazards model controlling for group (either of the tobacco smoking cohorts), age, gender, and degree of airflow obstruction (FEV1 percent predicted), elevated NT-proBNP (above the median of49 pg/mL) remained an independent predictor of mortality (HR = 2.19, 95% CI 1.07–4.46, p = 0.031). A similar trend of increased mortality was found when repeating the analysis across tertiles of increasing NT-proBNP (**Tables S4 and S5**). In this multivariate model, FEV1 percent predicted (per decile, HR = 0.75, 95%CI 0.65–0.87, p<0.001) was also significantly associated with mortality while cohort group, age, and gender were not associated with mortality.

**Figure 2:**
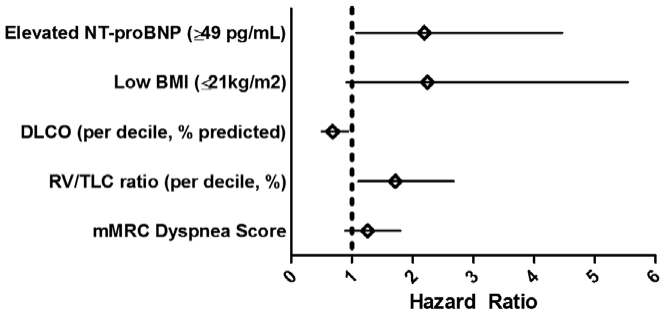
Multivariable Survival Analysis. Forrest plot showing survival hazard ratios. All variables were analyzed independently in a proportional hazard model incorporating group (patient cohort), age, gender, and degree of airflow obstruction (FEV1 percent predicted). Shown are estimated hazard ratio (diamonds) and 95% confidence intervals (whiskers). Due to missing values, the number of individuals differs slightly in each model, as follows: NT-proBNP (n = 779), Low BMI (n = 664), DLCO percent predicted (n = 665), RV/TLC ratio percent predicted (n = 569), mMRC dyspnea score (n = 761). *Definitions of abbreviations:* BMI = body mass index; DLCO = diffusion capacity for carbon monoxide; mMRC = modified Medical Research Council; NT-proBNP = N-terminal pro brain natriuretic peptide; RV/TLC = residual volume divided by total lung capacity.

When other factors known to have prognostic significance in COPD were assessed in the same proportional hazard model controlling for group, age, gender, and degree of airflow obstruction (FEV1 percent predicted), decreasing diffusion capacity (DLCO) (per decile, % predicted HR = 0.68, 95% CI 0.50–0.93, p = 0.015) and hyperinflation (RV/TLC ratio) (per decile, % HR = 1.71, 95% CI 1.10–2.67, p = 0.017) independently predicted mortality while low BMI (≤21 kg/m^2^) (HR = 2.24, 95% CI 0.91–5.54, p = 0.08), and mMRC dyspnea score (HR = 1.26, 95% CI 0.89–1.80, p = 0.19) did not **(** **Figure 2** **).**

## Discussion

In this study we have demonstrated that an elevated NT-proBNP level, independent of the degree of airflow obstruction, is a significant predictor of mortality in a large tobacco smoking cohort without known cardiovascular disease. Specifically, a NT-proBNP value of ≥49 pg/mL conferred a 2.2 fold increased risk of death independent of the severity of airflow obstruction and of other known prognostic indicators.

While NT-proBNP has not previously been reported to be a prognostic marker in tobacco smokers, elevated NT-proBNP has been associated with increased mortality in multiple other conditions, including CAD, CHF, and pulmonary arterial hypertension [11], [12], [13], [28]. We hypothesize that the increased mortality observed in our cohort is attributable to latent heart disease. This supposition is based upon literature that demonstrates that the increase in mortality associated with elevated NT-proBNP levels is concordant with an increase in cardiovascular morbidity and mortality [17], [18], [29]. However, the lack of diagnostic cardiac imaging or cause-specific mortality data in our cohort makes it impossible to substantiate this hypothesis.

In addition to NT-proBNP, other clinical characteristics known to predict mortality in tobacco smokers, including age, FEV1 percent predicted, hyperinflation (RV/TLC ratio), mMRC dyspnea scores, and low BMI (≤21 kg/m^2^), were found to be associated with mortality in our cohort in univariate analysis [30], [31], [32], [33], [34]. Notably, in multivariable analysis, NT-proBNP, FEV1, hyperinflation, and DLCO remained independent predictors of mortality. The finding that an elevated NT-proBNP is associated with mortality in tobacco users with a range of obstructive lung disease gives credence to the idea that this biomarker may be useful in assessing risk of death in those without worrisome traditional prognostic factors [5], [7].

The identification of heart disease in tobacco smokers can be diagnostically challenging as symptoms of cardiovascular disease and obstructive lung disease overlap [35]. Rutten and colleagues, in a study of stable COPD patients, found that a NT-proBNP value of >125 pg/mL was useful in detecting latent heart failure [15]. We found that 17.3% of our study subjects had a NT-proBNP value above this threshold. Thus, the results of our work, which show an increased risk of mortality, complement the diagnostic properties defined by others of NT-proBNP in this patient population at risk for cardiovascular disease. We propose that, in the context of additional studies, NT-proBNP could be used to risk-stratify tobacco smokers for diagnostic testing or therapeutic interventions, such as the prescription of beta blockers, which have recently been shown to reduce mortality in COPD patients both with and without cardiovascular disease [36], [37], [38].

The median value of NT-proBNP we found in our subjects (49 pg/mL) with a wide spectrum of obstructive lung disease and without known cardiovascular disease is not unexpected in comparison to other study groups. For example, in a healthy community dwelling cohort of adults >60 years old, the median NT-proBNP value for men was 40 pg/mL and was 78 pg/mL for women.[39] In our study cohort, the gender specific median NT-proBNP values are 40 pg/mL for men and 58 pg/mL for women. Watz et al. report a median NT-proBNP value of 67 pg/mL in their investigation of COPD patients without active heart disease, although the degree of airflow obstruction was greater in their study than ours (56% versus 69% percent predicted FEV1, respectively) [16]. Likewise, in accordance with the literature, we found in our study that increasing age, female gender, and decreasing BMI were independently associated with an elevated NT-proBNP [29], [40], [41], [42]. As reported by others, we found no correlation between severity of airflow obstruction as assessed by FEV1 and NT-proBNP levels [15], [16].

Although our study was based on a large prospective cohort of smokers with a wide spectrum of airflow obstruction and displayed good external validity in terms of corroborating well-described predictors of both elevated NT-proBNP and of mortality, there are certainly limitations worthy of consideration. We do not have detailed information regarding the presence of comorbid conditions in our subjects. In addition, we are unable to characterize the nature and severity of cardiovascular disease in our study patients. Finally, we lack information on exercise testing, which limits our ability to compare NT-proBNP to this important predictor of mortality either in isolation or in the form of the validated BODE (body mass index, airway obstruction, dyspnea, and exercise capacity) index [32].

In conclusion, we have shown in a tobacco smoking cohort without known cardiovascular disease that an elevated NT-proBNP is associated with increased mortality independent of airflow obstruction. The combined diagnostic and prognostic capabilities of NT-proBNP suggest that this readily available biomarker could be used to risk stratify those with or at-risk for COPD, a population with an increased prevalence of heart disease. Our findings should be confirmed in other cohorts of tobacco exposed patients and compared to established models of mortality risk assessment, such as the BODE index. If NT-proBNP is found to be a validated predictor of mortality, future prospective studies should assess the role of NT-proBNP to influence further diagnostic testing and to direct specific cardiovascular risk reduction inventions, such as beta blockade therapy, to impact quality of life and survival.

## Supporting Information

Table S1 Correlation analysis between NT-proBNP and demographics, lung function and renal function* (correlation coefficient, significance value).
(DOC)

Table S2 Survival Rates at Specific Follow Up Intervals. Survival rates are listed as percent survival (95% CI) for all subjects (All) and as dichotomized around the median NT-proBNP value (49 pg/mL) (High and Low NT-proBNP).
(DOC)

Table S3 Overall mortality by tertiles of NT-proBNP. Median (IQR) within each tertile of NT-proBNP and corresponding overall mortality rate.
(DOC)

Table S4 Univariate proportional hazard mortality analysis across increasing tertiles of NT-proBNP.
(DOC)

Table S5 Multivariate proportional hazard mortality analysis across increasing tertiles of NT-proBNP.
(DOC)
