# Supplementary material for: Elevated N-Terminal Pro-Brain Natriuretic Peptide Is Associated with Mortality in Tobacco Smokers Independent of Airflow Obstruction
Source: PLoS One. 2011 Nov 7;6(11):e27416. doi: 10.1371/journal.pone.0027416 (PMC3210169; doi:10.1371/journal.pone.0027416)
Supplement: Table S1 — Correlation analysis between NT-proBNP and demographics, lung function and renal function* (correlation coefficient, significance value). (DOC) [file pone.0027416.s001.doc]

Table S1. Correlation analysis between NT-proBNP and demographics, lung function and renal function* (correlation coefficient, significance value)

|  | Age (yrs) | BMI | FEV1 percent pred | FVC percent pred | FRC percent pred | DLCO percent pred | SCr |
| --- | --- | --- | --- | --- | --- | --- | --- |
| NT-proBNP | 0.27 (<0.0001) | -0.13 (0.004) | -0.008 (0.86) | -0.02 (0.64) | 0.09 (0.03) | -0.06 (0.21) | 0.03 (0.53) |

* *correlation performed using the nonparametric Spearman correlation, due to the non-normal distribution of NT-proBNP*
