# Supplementary material for: Elevated N-Terminal Pro-Brain Natriuretic Peptide Is Associated with Mortality in Tobacco Smokers Independent of Airflow Obstruction
Source: PLoS One. 2011 Nov 7;6(11):e27416. doi: 10.1371/journal.pone.0027416 (PMC3210169; doi:10.1371/journal.pone.0027416)
Supplement: Table S2 — Survival Rates at Specific Follow Up Intervals. Survival rates are listed as percent survival (95% CI) for all subjects (All) and as dichotomized around the median NT-proBNP value (49 pg/mL) (High and Low NT-proBNP). (DOC) [file pone.0027416.s002.doc]

Table S2. Survival Rates at Specific Follow Up Intervals. Survival rates are listed as percent survival (95% CI) for all subjects (All) and as dichotomized around the median NT-proBNP value (49 pg/mL) (High and Low NT-proBNP):

| Time period | Overall Survival (All) | High NT-proBNP | Low NT-proBNP |
| --- | --- | --- | --- |
| 6 months | 99 (98-100) | 99 (98-100) | 99 (97-100) |
| 12 months | 99 (98-99) | 99 (97-99) | 99 (97-100) |
| 24 months | 96 (94-98) | 95 (91-97) | 98 (95-99) |
| 36 months | 92 (88-95) | 90 (83-94) | 95 (89-98) |
