# Supplementary material for: Elevated N-Terminal Pro-Brain Natriuretic Peptide Is Associated with Mortality in Tobacco Smokers Independent of Airflow Obstruction
Source: PLoS One. 2011 Nov 7;6(11):e27416. doi: 10.1371/journal.pone.0027416 (PMC3210169; doi:10.1371/journal.pone.0027416)
Supplement: Table S3 — Overall mortality by tertiles of NT-proBNP. Median (IQR) within each tertile of NT-proBNP and corresponding overall mortality rate. (DOC) [file pone.0027416.s003.doc]

Table S3. Overall mortality by tertiles of NT-proBNP. Median (IQR) within each tertile of NT-proBNP and corresponding overall mortality rate:

| Tertile | NT-proBNP (pg/mL) | Percent Mortality |
| --- | --- | --- |
| 1 | 14 (7-22) | 3.4% |
| 2 | 49 (39-59) | 5.3% |
| 3 | 128 (94-200) | 7.2% |
