# Supplementary material for: Elevated N-Terminal Pro-Brain Natriuretic Peptide Is Associated with Mortality in Tobacco Smokers Independent of Airflow Obstruction
Source: PLoS One. 2011 Nov 7;6(11):e27416. doi: 10.1371/journal.pone.0027416 (PMC3210169; doi:10.1371/journal.pone.0027416)
Supplement: Table S4 — Univariate proportional hazard mortality analysis across increasing tertiles of NT-proBNP. (DOC) [file pone.0027416.s004.doc]

Table S4. Univariate proportional hazard mortality analysis across increasing tertiles of NT-proBNP

| NT-proBNP Tertile | Hazard Ratio (compared to lowest tertile of NT-proBNP) | 95% CI | P value |
| --- | --- | --- | --- |
| 1 | 1.0 (ref) |  | NA |
| 2 | 1.52 | 0.66-3.51 | 0.33 |
| 3 | 2.03 | 0.92-4.50 | 0.08 |
