# Supplementary material for: Elevated N-Terminal Pro-Brain Natriuretic Peptide Is Associated with Mortality in Tobacco Smokers Independent of Airflow Obstruction
Source: PLoS One. 2011 Nov 7;6(11):e27416. doi: 10.1371/journal.pone.0027416 (PMC3210169; doi:10.1371/journal.pone.0027416)
Supplement: Table S5 — Multivariate proportional hazard mortality analysis across increasing tertiles of NT-proBNP. (DOC) [file pone.0027416.s005.doc]

Table S5. Multivariate proportional hazard mortality analysis across increasing tertiles of NT-proBNP*

| NT-proBNP Tertile | Hazard Ratio (compared to lowest tertile of NT-proBNP) | 95% CI | P value |
| --- | --- | --- | --- |
| 1 | 1.0 (ref) |  | NA |
| 2 | 1.43 | 0.61-3.34 | 0.41 |
| 3 | 2.01 | 0.86-4.69 | 0.11 |

** controlling for registry, age, gender, and degree of airflow obstruction*
